# Molecular Characterization of Class A-ESBLs, Class B-MBLs, Class C-AmpC, and Class D-OXA Carbapenemases in MDR Acinetobacter baumannii Clinical Isolates in a Tertiary Care Hospital, West Bengal, India

**DOI:** 10.7759/cureus.43656

**Published:** 2023-08-17

**Authors:** Langamba A Longjam, Dechen C Tsering, Dipmala Das

**Affiliations:** 1 Microbiology, Sikkim Manipal Institute of Medical Sciences, Sikkim Manipal University, Gangtok, IND; 2 Microbiology, IQ City Medical College and Hospital, Durgapur, IND

**Keywords:** real-time pcr, carbapenem hydrolyzing class d β-lactamases, acinetobacter calcoaceticus baumannii complex, primer, ampc cephalosporinase, acinetobacter baumannii, multidrug-resistant acinetobacter, oxa-51, metallo β-lactamases, extended-spectrum β-lactamases (esbls)

## Abstract

Background

*Acinetobacter calcoaceticus baumannii* (ACB) complex has become a major concern nowadays because of its increasing involvement in several severe infections associated with catheter-related bloodstream and urinary tract infections, ventilator-associated pneumonia, cerebrospinal shunt-related meningitis, and wound infections. Multiple drug-resistant (MDR) ACB cases have been described with an increasing trend where at least it is resistant to a minimum of three antimicrobial groups. The mortality rate associated with *A. baumannii *is significantly higher than all *Acinetobacter* spp. isolates with the most prevalence seen in India and Thailand. The rapid spread of high resistance to most potent antimicrobial drugs is due to its ability to incorporate resistance determinants despite multifactorial reasons such as alteration in permeability of cell membrane by either losing expression of outer membrane porins or excess production of efflux pumps. This study aims to characterize resistance determinants responsible for MDR at the genetic level and emphasizes the use of genotyping in routine diagnosis as genotype analysis is reliable and valid.

Methodology

A total of 289 ACB complex clinical isolates were included in this study. The study for species-level identification of *A. baumannii *was conducted at the Department of Microbiology, IQ City Medical College Hospital, Durgapur, West Bengal. In addition, the detection of encoded genes associated with class A-extended spectrum beta-lactamases (i.e., *CTX-M*, *KPC*, *SHV*, and *TEM* genes), class B-metallo-β-lactamases (i.e., *IMP*, *NDM*, and *VIM* genes), Class C-AmpC cephalosporinase, and classD-OXA carbapenemases (i.e., *bla*_OXA-10/11_, *bla*_OXA-24_, *bla*_OXA-48_, *bla*_OXA-58_, *bla*_OXA-143_, and *bla*_OXA-235_ was done using real-time polymerase chain reaction.

Results

All 289 non-repetitive ACB complex clinical isolates were confirmed as *A. baumannii*, of which 277 (96%) isolates were MDR. There were no findings of *bla_CTX-M_*, *bla_KPC_*, *bla_SHV_*, *bla_TEM_*, *bla_IMP_*, *bla_VIM_*, *bla_AmpC_*, *bla_OXA-10/11_*, *bla_OXA-24_*, *bla_OXA-48_*, *bla_OXA-58_*, *bla_OXA-143_*, and *bla_OXA-235_* genes in our study. However, there were four (1.44%) positive findings of the *bla_NDM_* gene. All MDR isolates (n = 277) were positive for the *bla_OXA-51_* gene. In addition, *bla_OXA-23_* was positive in 269 (97.12%) isolates.

Conclusions

The oxacillinase production corresponding to *bla*_OXA-23_ and* bla*_OXA-51_ were the most prevalent antibiotic resistance determinants among MDR *A. baumannii *in our study. Four (1.44%) isolates had the multiple genes *bla*_OXA-51_, *bla*_OXA-23_, and *bla*_NDM_ that shows the coexistence of diverse genetic elements involved in MDR *A. baumannii*, resulting in total resistance except for a few potent drugs such as colistin and tigecycline. Genotyping is helpful in determining the contribution of the isolates in understanding their association with encoded genes, which, in turn, helps in designing effective surveillance and control strategies in the management of such MDR isolates.

## Introduction

*Acinetobacter calcoaceticus baumannii* (ACB) complex has become a major concern nowadays because of its increasing involvement in several severe infections associated with catheter-related bloodstream and urinary tract infections, ventilator-associated pneumonia, cerebrospinal shunt-related meningitis, and wound infections [1]. It accounted for 1% to 2% of all bloodstream infections in a hospital-based study of developed countries, i.e., 52 hospitals located in the United States. They are typically associated with intravascular catheters (15.3%) and the respiratory tract (12.9%). Overall, 63% of the infections were associated with *A. baumannii*. The mortality rate associated with *A. baumannii* (36.8%) was significantly higher than those with *A. nosocomialis* (16.4%) and *A. pittii* (13.0%) [2]. Moreover, 1.2% to 87% of all *Acinetobacter* isolates were multiple drug resistant (MDR) with most prevalence seen in India and Thailand [3]. The MDR ACB is defined as isolates that provide resistance to a minimum of three antimicrobial groups [4]. This results in high rates of morbidity and mortality in the healthcare system due to its wider resistance to the most potent antimicrobial drugs. Moreover, there are reports of *A. baumannii* being acquired through the community [5].

The recently advanced whole-genome sequencing analysis has suggested the association of the rapid spread of MDR *A.* *baumannii* with the ability to incorporate resistance determinants [6-8]. Resistance to potent antibiotics in *A. baumannii* is primarily mediated by the genes that encode β-lactamase belonging to class A, B, and C AmpC cephalosporinase and D carbapenem-hydrolyzing β-lactamases despite multifactorial reasons such as alteration in the permeability of cell membrane by either losing expression of outer membrane porins or excess production of efflux pumps.

Our previous phenotypic study [4] clearly demonstrated that the ACB complex clinical isolates were resistant to the most potent drugs and further suggested its identification at the species level and association with resistance determinants at the genetic level. Therefore, this study was conducted to determine the incidence rate of *A. baumannii *from ACB complex clinical isolates and their resistance determinants responsible for β-lactamase in association with MDR *A. baumannii *as genotype analysis is reliable and valid.

## Materials and methods

Bacterial isolates

The study for species-level identification of *A. baumannii* from previously phenotypically identified non-repetitive clinical isolates of 289 ACB complex [4] was conducted at the Department of Microbiology, IQ City Medical College Hospital, Durgapur, West Bengal.

Bacterial DNA preparation and identification of *A. baumannii*

The commercially available spin column method of bacterial DNA isolation kit of GSure® Bacterial Genomic DNA Isolation Kit (GCC Biotech®, West Bengal, India) was used to prepare bacterial DNA isolates from a pure culture grown in peptone water and then kept at -20°C for further use.

The bacterial DNA extracts were identified for their species level, i.e., *A. baumannii* using real-time polymerase chain reaction (PCR) (CFX96™, Bio-Rad Laboratories, Inc.) for its further use in the genotypic analysis of certain antibiotic resistance determinants. A pair of forward and reverse primers that were specific to *A. baumannii* was used to amplify the target DNA. The hold stage was performed at the corresponding temperature of 50°C for five minutes. Consecutively, a corresponding temperature of 95°C was also applied for five minutes. In total, 45 cycles of continuous polymerization occurred at a corresponding temperature of 95°C for 15 seconds. Consecutively, a corresponding temperature of 60°C was also applied for 30 seconds.

Detection of encoded genes associated with drug resistance by real-time PCR

Multiplex PCR and uniplex PCR of certain antibiotic resistance determinants were analyzed as described below.

Multiplex Real-Time PCR Detection Assay

Multiplex real-time PCR was done to detect the class A group of β-lactamase, i.e., extended-spectrum (ESBLs) genes such as *TEM*, *SHV*, and *CTX-M* genes.

For this, 5 µL of extracted DNA template was added to a 20 µL reagent mixture (containing 12.5 µL master mix of Taq DNA polymerase, dNTPs mix, PCR buffer; 1 µL of internal control primer probe; 1 µL of internal control DNA; 3 µL of the respective primers, as shown in Table 1; and 2.5 µL of PCR-grade water). The initial denaturation of the above-mentioned genes was performed at a corresponding temperature of 95°C for 10 minutes. Thereafter, 40 cycles of further denaturation at a corresponding temperature of 95°C for 15 seconds. Annealing and extension were done at a corresponding temperature of 60°C for 30 seconds.

**Table 1:** Primers used in the present study.

Target genes	Primer sequences (5’ to 3’)		Amplicon size (bp)
	Forward	Reverse	
bla_AmpC_	TAAACACCACATATGTTCCG	ACTTACTTCAACTCGCGACG	663
bla_CTX-M_	TTAGGAARTGTGCCGCTGYA	CGATATCGTTGGTGGTRCCAT	688
bla_IMP_	GAAGGYGTTTATGTTCAT	GTAMGTTTCAAGAGTGAT	587
bla_KPC_	GTATCGCCGTCTAGTTCTGC	GGTCGTGTTTCCCTTTAGCC	637
bla_NDM_	GGTTTGGCGATCTGGTTTTC	CGGAATGGCTCATCACGATC	623
bla_OXA-10/11_	TCAACAAATCGCCAGAGAAG	TCCCACACCAGAAAAACCAG	276
bla_OXA-23_	GATCGGATTGGAGAACCAGA	ATTTCTGACCGCATTTCCAT	501
bla_OXA-24_	GGTTAGTTGGCCCCCTTAAA	AGTTGAGCGAAAAGGGGATT	246
bla_OXA-48_	TTGGTGGCATCGATTATCGG	GAGCACTTCTTTTGTGATGGC	744
bla_OXA-51_	TAATGCTTTGATCGGCCTTG	TGGATTGCACTTCATCTTGG	353
bla_OXA-58_	AAGTATTGGGGCTTGTGCTG	CCCCTCTGCGCTCTACATAC	599
bla_OXA-143_	TGGCACTTTCAGCAGTTCCT	TAATCTTGAGGGGGCCAACC	149
bla_OXA-235_	TTGTTGCCTTTACTTAGTTGC	CAAAATTTTAAGACGGATCG	768
bla_SHV_	AGCCGCTTGAGCAAATTAAAC	ATCCCGCAGATAAATCACCAC	713
bla_TEM_	CATTTCCGTGTCGCCCTTATTC	CGTTCATCCATAGTTGCCTGAC	800
bla_VIM_	GGGAGCCGAGTGGTGAGT	GGCACAACCACCGTATAG	519

A second multiplex PCR was done to detect class B β-lactamase, i.e., metallo β-lactamase (MBLs) genes such as *NDM*, *IMP*, *VIM*, and *KPC *genes.

For this, a 5 µL extracted DNA template was added to a 20 µL reagent mixture (containing 12.5 µL master mix of Taq DNA polymerase, dNTPs mix, PCR buffer; 1 µL of internal control primer probe; 1 µL of internal control DNA, 4 µL of the respective primers, as shown in Table 1; and 1.5 µL of PCR-grade water). The initial denaturation of the above-mentioned genes was performed at a corresponding temperature of 95°C for 10 minutes. Thereafter, 45 cycles of further denaturation at a corresponding temperature of 95°C for five seconds. Annealing and extension were done at a corresponding temperature of 60°C for one minute.

A third multiplex PCR was done to detect the class D group of β-lactamase, i.e., carbapenem hydrolyzing (CHDLs) genes such as *OXA-58*, *OXA-51*, *OXA-48*, and *OXA-23*. The components and conditions for the thermocycle were identically performed as the above-mentioned second multiplex PCR except for the corresponding primers, as shown in Table 1.

Uniplex PCR

Uniplex PCR was done to detect the class C AmpC cephalosporinase gene and the CHDL genes such as *OXA-24*, *OXA-143*, and *OXA-235*.

For this, a 10 µL extracted DNA template was added to a 15 µL reagent mixture (containing 10 µL master mix of Taq DNA polymerase, dNTPs mix, PCR buffer, molecular grade water, and 5 µL of the respective primers, as shown in Table 1). The condition for thermocycle of the above-mentioned genes was Taq enzyme activation or the hold stage at a temperature of 95°C for about 15 minutes. Thereafter, 35 cycles of further denaturation at a corresponding temperature of 95°C for 20 seconds. Annealing was done at a corresponding temperature of 60°C for 20 seconds, followed by extension at a corresponding temperature of 72°C for 20 seconds.

Data analysis

A database for genotypic data collection and analysis was formed by using Microsoft Excel 2013 and descriptive analysis was done for the respective data.

## Results

**Figure 1:**
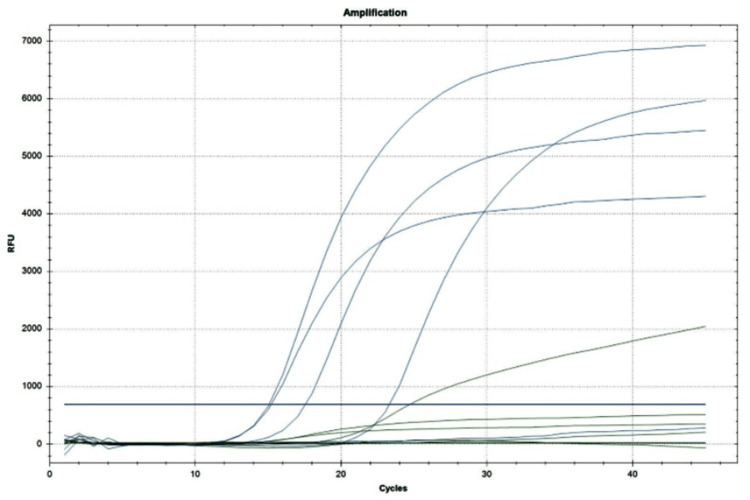
A. baumannii PCR determination using the FAM fluorophore dye for detection of target gene 1 specific for A. baumannii at the source wavelength of 470 nm and detection wavelength of 510 nm and the HEX fluorophore dye for the detection of the internal control gene at the source wavelength of 530 nm and detection wavelength of 555 nm. The threshold cycle value is ≤40 for both the target as well as the internal control gene. The limit of detection was found to be DNA 10 copies/reaction.

All 289 non-repetitive ACB complex clinical isolates were confirmed as *A. baumannii *by performing real-time PCR analysis (Figure 1). Out of the 289 confirmed *A. baumannii*, 277 (96%) isolates were MDR. These MDR *A. baumannii* isolates were used for further molecular analysis.

Regarding the identification of the class A group of β-lactamase, i.e., ESBLs such as *CTX-M*, *KPC*, *SHV*, and *TEM*,there were no findings of *bla*_CTX-M_, *bla*_KPC_, *bla*_SHV_, and *bla*_TEM_ genes in our study, as shown in Table 2.

**Table 2:** Distribution of different types of β-lactamases among MDR A. baumannii. ESBL = extended-spectrum β-lactamase; MBL = metallo-β-lactamase; MDR = multiple drug resistant

ESBL type	Total number of isolates (N) = 277
bla_CTX-M_ (%)	0 (0)
bla_KPC _(%)	0 (0)
bla_SHV _(%)	0 (0)
bla_TEM _(%)	0 (0)
MBL type	
bla_IMP _(%)	0 (0)
bla_NDM _(%)	4 (1.44)
bla_VIM_ (%)	0 (0)
AmpC type	
bla_AmpC _(%)	0 (0)
OXA type	
bla_OXA-10/11_(%)	0 (0)
bla_OXA-23 _(%)	269 (97.12)
bla_OXA-24_ (%)	0 (0)
bla_OXA-48 _(%)	0 (0)
bla_OXA-51 _(%)	277 (100)
bla_OXA-58 _(%)	0 (0)
bla_OXA-143 _(%)	0 (0)
bla_OXA-235 _(%)	0 (0)

**Figure 2:**
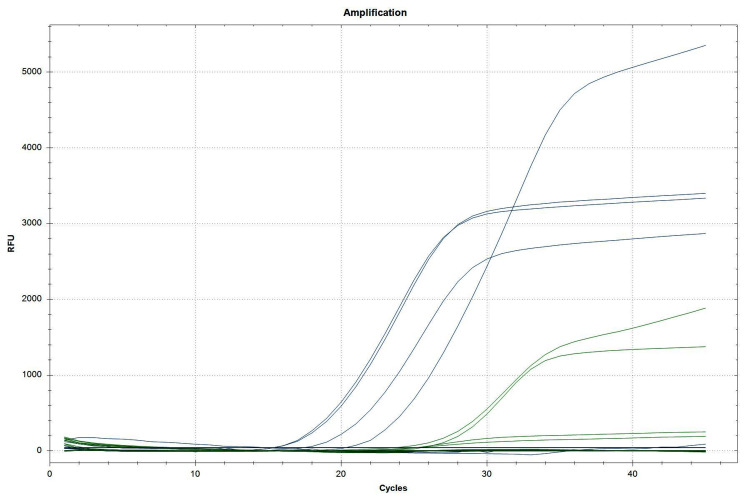
NDM target gene polymerase chain reaction determination using the FAM fluorophore dye for the detection of the NDM gene and Cy5.5 fluorophore dye for the detection of the internal control. The threshold cycle value is ≤40 for both the target as well as the internal control gene.

Regarding the identification of class B β-lactamase, i.e., MBLs such as *IMP*, *NDM*, and *VIM* genes, there were no findings of *bla*_IMP_ and *bla*_VIM_ genes in our study, as shown in Table 2. However, there were four (1.44%) positive findings of the *bla*_NDM_ gene, as shown in Table 2 and Figure 2.

Regarding the identification of class C AmpC cephalosporinase, there were no findings of the AmpC (*bla*_AmpC_) gene in our study, as shown in Table 2.

**Figure 3:**
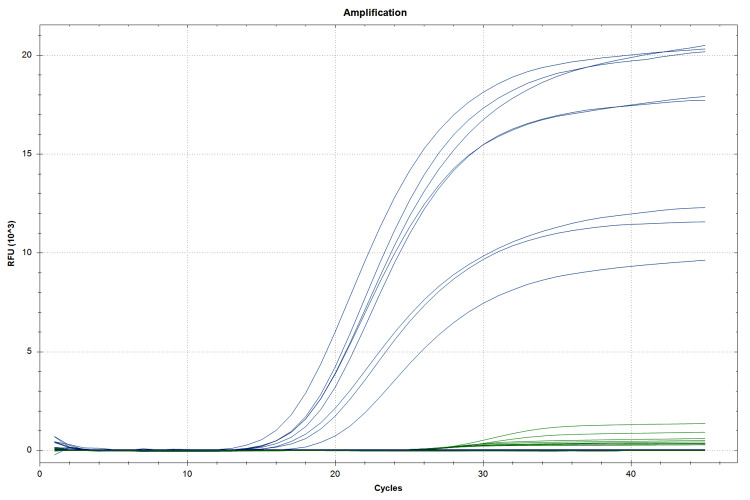
OXA-51 target gene polymerase chain reaction determination using the FAM fluorophore dye for the detection of the OXA-51 gene and Cy5.5 fluorophore dye for the detection of the internal control. The threshold cycle value is ≤40 for both the target as well as the internal control gene.

**Figure 4:**
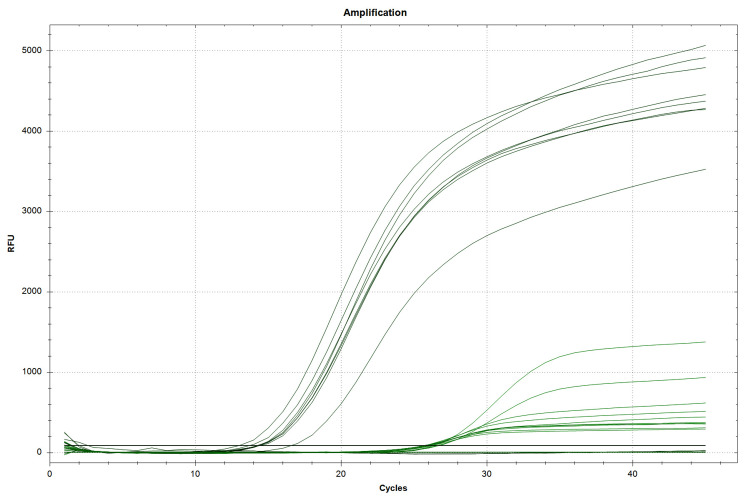
OXA-23 target gene polymerase chain reaction determination using the HEX fluorophore dye for the detection of the OXA-23 gene and Cy5.5 fluorophore dye for the detection of the internal control. The threshold cycle value is ≤40 for both the target as well as the internal control gene.

As shown in Table 2 and Figure 3, all the isolates (n = 277) were positive for the *bla_OXA-51_* gene. In addition, *bla*_OXA-23_ was positive in 269 (97.12%) isolates, as shown in Table 2 and Figure 4. However, there were no findings of *bla*_OXA-235_, *bla*_OXA-143_, *bla*_OXA-58_, *bla*_OXA-48_, *bla*_OXA-24_, and *bla*_OXA-10/11_.

Thus, the oxacillinase production corresponding to *bla*_OXA-23_ and *bla*_OXA-51_ were the most prevalent antibiotic resistance determinants among MDR *A. baumannii*. Four (1.44%) isolates had the multiple genes *bla*_OXA-51_, *bla*_OXA-23_, and *bla*_NDM_ that shows the coexistence of diverse genetic elements involved in MDR *A. baumannii*, resulting in total resistance except for a few potent drugs such as colistin and tigecycline.

## Discussion

There was no significant variability among the data generated in the phenotypic and genotypic analyses but there was a comparative difference qualitatively. Both data have their associated advantages and disadvantages as bacterial phenotypic expression is affected by external physical factors. Therefore, the epidemiological typing was not enough by phenotypic analysis. Phenotypic tests are easy to perform and inexpensive compared to genotypic tests, whereas genotypic analysis is reliable and valid.

Our study reveals the high prevalence of *bla*_OXA-51_ (100%) and *bla*_OXA-23_ (97.12%) genes in MDR *A. baumannii* which shows that these genes can confer resistance to multiple potent antibiotics. According to several reports, the association of the insertion sequence ISAba1 element in conferring the carbapenemase activity of *bla*_OXA-51_ is important [5]. Therefore, the involvement of the *bla*_OXA-51_ gene alone in conferring resistance to multiple potent antibiotics is uncertain as there was no identification of the ISAba1 element through PCR in our study.

However, the detection of *bla*_OXA-51_ in all isolates supports the use of the *bla*_OXA-51_ gene as a surrogate marker of *A. baumannii* identification, as reported by different studies [9-13]. The *bla*_OXA-51 _gene has been shown to be widely associated with the ACB complex collected from various geographic regions [14-18]. This means that some of the members of the phenotypically identified ACB complex isolates possess the *bla*_OXA-51_ gene. Moreover, in the study by Teixeira et al., there was the presence of plasmid-encoded *bla*_OXA-51_ in *A. nosocomialis* clinical isolates [19].

Our study reveals the 97.12% presence of *bla*_OXA-23_ in MDR *A. baumannii*. In the study by Vijayakumar et al., 98% of *A. baumannii* clinical isolates showed the presence of *bla*_OXA-23_, highlighting that *bla*_OXA-23_ is also a predominantly antibiotic resistance determinant in *A. baumannii *[20].

The MBLs are less commonly identified in *A. baumannii* than oxacillinases, but their hydrolytic activities toward carbapenems except the monobactam aztreonam are significantly more potent (100 to 1,000 fold) [21]. In our study, there were no findings of *bla*_VIM_ and *bla*_IMP_ genes which are the commonly isolated MBL genes if present. However, four (1.44%) positive findings of the *bla*_NDM _gene in our study show the possibilities of various risk factors and their correlation with other genes such as *bla*_OXA-51_ and *bla*_OXA-23_ substantiates the coexistence of diverse genetic elements involved in MDR *A. baumannii*, providing total resistance for potent drugs.

In our study, there were no findings of *bla*_CTX-M_, *bla*_KPC_, *bla*_SHV_, *bla*_TEM_, *bla*_AmpC_, *bla*_OXA-235_, *bla*_OXA-143_, *bla*_OXA-58_, *bla*_OXA-48_, *bla*_OXA-24_, and *bla*_OXA-10/11 _genes. This study has limitations of a few small isolates and being a single-centered study. The association of insertion sequence with the overexpression of antibiotic resistance genes is yet to be explored in further studies. Moreover, the possibilities of diverse antibiotic resistance mechanisms such as alteration in the permeability of cell membrane, permeability by a decrease in the downregulation of outer membrane proteins, or upregulation of efflux pumps also exist.

## Conclusions

This study shows that *bla*_OXA-51_ and *bla*_OXA-23_ are the most common antibiotic resistance determinants among MDR *A. baumannii* clinical isolates. The prevalence of multiple *bla*_OXA-51_, *bla*_OXA-23_, and *bla*_NDM_ genes in isolates pose a threat to the healthcare community for outbreaks of total resistance to potent drugs and limit the therapeutic options. Moreover, there is an emergence of the *bla*_NDM _gene involved in MDR *A. baumannii *clinical isolates that would show the serious problem of antimicrobial therapy. This shows the necessity of establishing relevant infection control practices or policies by respective stakeholders in response to the epidemiological data.
